# Influence of Age at Diagnosis on Clinical Disease Parameters in Wild-Type Transthyretin Amyloid Cardiomyopathy

**DOI:** 10.1016/j.jacasi.2025.06.003

**Published:** 2025-07-29

**Authors:** Naoto Kuyama, Yasuhiro Izumiya, Seiji Takashio, Hiroki Usuku, Akihisa Tabira, Masanobu Ishii, Masafumi Kidoh, Seitaro Oda, Shinsuke Hanatani, Yasushi Matsuzawa, Eiichiro Yamamoto, Toshinori Hirai, Mitsuharu Ueda, Kenichi Tsujita

**Affiliations:** a Department of Cardiovascular Medicine, Graduate School of Medical Sciences, Kumamoto University, Kumamoto, Japan; b Department of Medical Information Science, Graduate School of Medical Sciences, Kumamoto University, Kumamoto, Japan; c Department of Diagnostic Radiology, Graduate School of Medical Sciences, Kumamoto University, Kumamoto, Japan; d Department of Neurology, Graduate School of Medical Sciences, Kumamoto University, Kumamoto, Japan; e Center for Metabolic Regulation of Healthy Aging, Faculty of Life Sciences, Kumamoto University, Kumamoto, Japan

**Keywords:** age at diagnosis, ATTR-CM, biomarkers, cardiac imaging, sex differences, transthyretin amyloid cardiomyopathy

Wild-type transthyretin (TTR) amyloid cardiomyopathy (ATTRwt-CM) is an age-related disease characterized by amyloid deposition in the myocardium. Advances in diagnostic techniques^1^^,^^2^ have led to increased diagnoses, yet the impact of age at diagnosis on clinical parameters is poorly understood. Although ATTRwt-CM is more common in older individuals,^3^^,^^4^ some younger patients present with advanced disease, suggesting age alone does not dictate disease severity. This study aimed to explore the association between the age at diagnosis and disease parameters of ATTRwt-CM to enable a more accurate interpretation of each parameter in patients with ATTRwt-CM.

This single-center retrospective study included 307 patients diagnosed with ATTRwt-CM at Kumamoto University Hospital between November 28, 2013 and September 7, 2024. Patients were referred from referrals to our tertiary center and diagnosed based on current noninvasive and invasive criteria. All 307 patients were confirmed to have no *TTR* mutations through TTR genetic testing. Early-diagnosis ATTRwt-CM was defined as diagnosis before 75 years, aligning with the definition of late-stage elderly individuals in Japan. Clinical demographics, laboratory findings, echocardiography, cardiac magnetic resonance, bone scintigraphy, and right heart catheterization data were collected. Contrast-enhanced and noncontrast cardiac magnetic resonance were performed in 155 and 175 patients, respectively. Right heart catheterization is performed simultaneously as part of daily clinical practice, including concurrent myocardial biopsy for definitive diagnosis in 207 patients. Cardiac amyloid load was quantified using right ventricular septal biopsy specimens.^5^ Congo red staining identified amyloid deposition, which was analyzed with ImageJ (National Institutes of Health). The amyloid load (%) was calculated as the amyloid deposition area relative to the total myocardial area × 100. Continuous variables were analyzed using Student’s *t*-tests or Wilcoxon signed-rank tests and categorical variables with chi-square tests. Pearson correlation coefficients assessed relationships between age and disease parameters. Logistic regression identified predictors of early-diagnosis ATTRwt-CM. The study protocol was approved by the Human Ethics Committee of Kumamoto University (approval no. 1590).

The study cohort comprised 307 patients, with 116 (38%) diagnosed before 75 years (early-diagnosis group: mean age, 70.2 ± 3.4 years) and 191 (62%) diagnosed at or after 75 years (late-diagnosis group: mean age, 80.0 ± 3.5 years). The early-diagnosis group had a significantly higher proportion of male patients (98% vs 82%, *P* < 0.01) and better renal function. Late-diagnosis patients had higher NYHA functional class, prior heart failure hospitalizations, and hypertension, whereas carpal tunnel syndrome and lumbar spinal canal stenosis were more common in early-diagnosis patients. Late-diagnosis patients exhibited higher high-sensitivity cardiac troponin T (hs-cTnT) and B-type natriuretic peptide (BNP) levels (*P* < 0.01) and increased left atrial volume index and E/eʹ ratio. However, left ventricular ejection fraction (LVEF), global longitudinal strain, native T_1_, extracellular volume, heart-to-contralateral (H/CL) ratio, and hemodynamic parameters, including pulmonary capillary wedge pressure, pulmonary artery pressure, and cardiac index, did not differ significantly between groups. Multivariable logistic regression analysis identified male sex (OR: 9.60; 95% CI: 2.17-42.4; *P* < 0.01), lumbar spinal canal stenosis, and higher estimated glomerular filtration rate (eGFR) (OR: 1.03; 95% CI: 1.01-1.05; *P* < 0.01, per 1 mL/min/1.73 m^2^) as independent predictors of early-diagnosis disease even after adjusting NYHA functional class III, prior heart failure hospitalizations, hypertension, carpal tunnel syndrome, diuretic use, and lower hs-cTnT and BNP levels. A sensitivity analysis using linear regression analysis for age at diagnosis showed results consistent with those obtained from logistic regression analysis.

Age at diagnosis demonstrated a statistically significant but modest correlation with key disease biomarkers and imaging measures (Figure 1). Positive correlations were observed between age at diagnosis and log-transformed hs-cTnT (*r* = 0.228; *P* < 0.01) as well as BNP (*r* = 0.306; *P* < 0.01). Conversely, a negative correlation was noted between age at diagnosis and eGFR (*r* = 0.347; *P* < 0.01) and H/CL ratio (*r* = 0.155; *P* = 0.02). Notably, structural and functional cardiac parameters, including LVEF, global longitudinal strain, native T_1_, extracellular volume, and right heart catheterization data, demonstrated minimal age-related variation, supporting their reliability across age groups for disease severity assessment.

**Figure 1:**
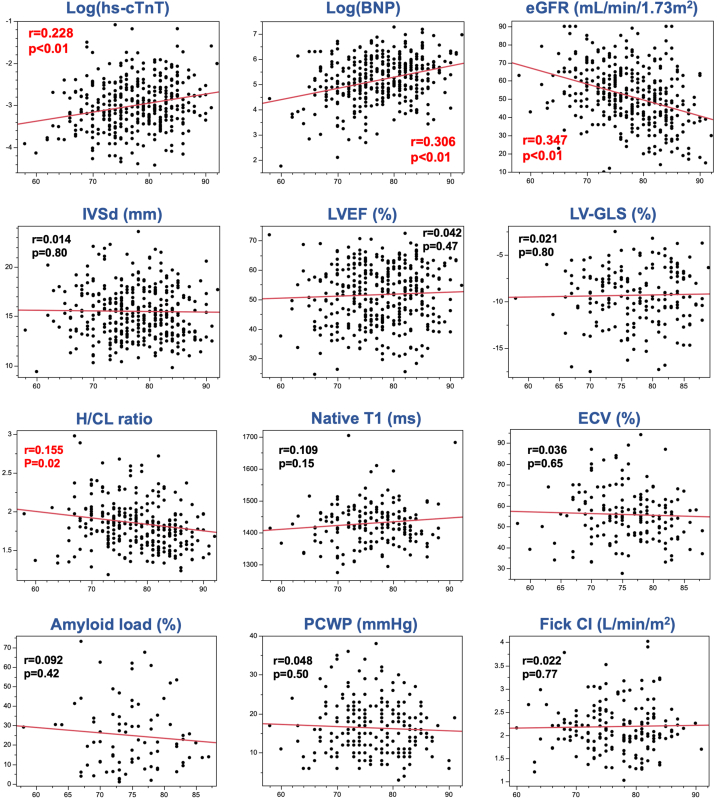
Correlation Between Age and Clinical Parameters This figure illustrates the correlations between age at diagnosis and various clinical parameters in patients with wild-type transthyretin amyloid cardiomyopathy. Scatter plots display relationships between age at diagnosis (x-axis) and biomarkers, structural parameters, imaging markers, and hemodynamic measures. Modest but statistically significant positive correlations were observed with high-sensitivity cardiac troponin T (hs-cTnT) and B-type natriuretic peptide (BNP), whereas estimated glomerular filtration rate (eGFR) and heart-to-contralateral ratio (H/CL) ratio showed significant negative correlations. Other imaging and functional parameters demonstrated minimal or no association with age. These results highlight the variable age-dependence among disease markers and underscore the potential stability of imaging and functional parameters across age groups. CI = cardiac index; ECV = extracellular volume; IVSd = interventricular septal thickness in diastole; LVEF = left ventricular ejection fraction; LV-GLS = left ventricular global longitudinal strain; PCWP = pulmonary capillary wedge pressure.

This study provides new insights into the influence of age on ATTRwt-CM presentation. Whereas male sex strongly predicted early diagnosis, disease severity at diagnosis was comparable between early- and late-diagnosis groups. The modest but statistically significant age-related increases in hs-cTnT and BNP suggest that age may have a partial influence on biomarker levels, possibly reflecting age-associated physiological changes in cardiac stress,^6^^,^^7^ neurohormonal activity,^8^ and renal function.^9^ In contrast, imaging and functional parameters demonstrated minimal correlation with age, indicating their potential as more stable and reliable indicators of disease severity across a broad age spectrum. This finding highlights the need for a multimodal diagnostic approach that integrates biomarkers with imaging and functional data to ensure the accurate evaluation of ATTRwt-CM, irrespective of patient age.

Our findings demonstrated that male sex was strongly associated with early-diagnosis, suggesting a potential sex-specific predisposition for the development of ATTRwt-CM at a younger age. Previous reports have indicated that female patients with ATTRwt-CM had thinner left ventricular wall thickness, preserved LVEF, and weaker cardiac uptake of the bone scintigraphy tracer compared with male patients,^10^^,^^11^ which may contribute to delayed diagnosis in female patients, though further investigation is needed to elucidate these mechanisms.

This study had several limitations. This was a single-center retrospective study, limiting generalizability. Given the lack of precise data on time-to-diagnosis, the age of diagnosis may not necessarily reflect the actual onset of disease, suggesting that future studies with longitudinal follow-up would better capture disease onset. Furthermore, given that this study spans a long observation period, variations in diagnostic criteria and time to diagnosis may have affected the results. Therefore, future analyses stratifying patients based on the diagnostic era should be considered. With advances in diagnostic criteria and increased physician awareness, there has been a trend toward earlier detection of ATTRwt-CM. This shift may have contributed to the observed differences in age at diagnosis across the study period, potentially influencing the comparison between early- and late-diagnosed groups.

In conclusion, age and sex significantly influence ATTRwt-CM presentation, with male sex strongly associated with early-onset disease. Despite age differences, disease severity at diagnosis remains similar across groups. Biomarkers may require age-specific interpretation, but imaging and functional parameters are reliable across all ages, emphasizing the need for comprehensive diagnostic approaches.

## Funding Support and Author Disclosures

This study was supported by Japan Society for the Promotion of Science Grant-in-Aid for Scientific Research (grant no. 23K19595 for N.K. and no. 22K08134 for Y.I.). Dr Tsujita has reported that he has received significant research grants from AMI Co Ltd, Bayer Yakuhin Ltd, Bristol-Myers KK, EA Pharma Co Ltd, Mochida Pharmaceutical Co Ltd; scholarship funds from AMI Co Ltd, Bayer Yakuhin Ltd, Boehringer Ingelheim Japan, Chugai Pharmaceutical Co Ltd., Daiichi-Sankyo Co Ltd, Edwards Lifesciences Corporation, Johnson & Johnson KK, Ono Pharmaceutical Co Ltd, Otsuka Pharmaceutical Co Ltd, Takeda Pharmaceutical Co Ltd; and honoraria from Amgen KK, Bayer Yakuhin Ltd, Daiichi-Sankyo Co Ltd, Kowa Pharmaceutical Co Ltd, Novartis Pharma KK, Otsuka Pharmaceutical Co Ltd, Pfizer Japan Inc; and he belongs to the endowed departments donated by Abbott Japan Co Ltd, Boston Scientific Japan KK, Fides-one Inc, GM Medical Co Ltd, ITI Co Ltd, Kaneka Medix Co Ltd, Nipro Corporation, Terumo Co Ltd, Abbott Medical Co Ltd, Cardinal Health Japan, Fukuda Denshi Co Ltd, Japan Lifeline Co Ltd, Medical Appliance Co Ltd, Medtronic Japan Co Ltd. All other authors have reported that they have no relationships relevant to the contents of this paper to disclose.
